# Ischemic heart injury leads to HIF1-dependent differential splicing of CaMK2γ

**DOI:** 10.1038/s41598-021-92426-2

**Published:** 2021-06-23

**Authors:** Allison Lesher Williams, Chad B. Walton, Blake Pinell, Vedbar S. Khadka, Brandyn Dunn, Katie Lee, M. C. Therese Anagaran, Abigail Avelar, Ralph V. Shohet

**Affiliations:** 1 Center for Cardiovascular Research, John A. Burns School of Medicine, University of Hawaii, 651 Ilalo St. BSB 311, Honolulu, HI 96813 USA; 2 Bioinformatics Core, John A. Burns School of Medicine, University of Hawaii, Honolulu, HI USA

**Keywords:** Transcriptomics, Gene expression, RNA sequencing

## Abstract

Ischemic heart disease is a leading cause of heart failure and hypoxia inducible factor 1 (HIF1) is a key transcription factor in the response to hypoxic injury. Our lab has developed a mouse model in which a mutated, oxygen-stable form of HIF1α (HIF-PPN) can be inducibly expressed in cardiomyocytes. We observed rapid cardiac dilation and loss of contractility in these mice due to lower expression of excitation–contraction coupling genes and reduced calcium flux. As alternative splicing plays an underappreciated role in transcriptional regulation, we used RNA sequencing to search for splicing changes in calcium-handling genes of HIF-PPN hearts and compared them to previous sequencing data from a model of myocardial infarction (MI) to select for transcripts that are modified in a pathological setting. We found overlap between genes differentially expressed in HIF-PPN and post-MI mice (54/131 genes upregulated in HIF-PPN hearts at 1 day and/or 3 days post-MI, and 45/78 downregulated), as well as changes in alternative splicing. Interestingly, calcium/calmodulin dependent protein kinase II, gamma (*CAMK2G*) was alternatively spliced in both settings, with variant 1 (v1) substantially decreased compared to variants 2 (v2) and 3 (v3). These findings were also replicated in vitro when cells were transfected with HIF-PPN or exposed to hypoxia. Further analysis of CAMK2γ protein abundance revealed only v1 was detectable and substantially decreased up to 7 days post-MI. Rbfox1, a splicing factor of *CAMK2G*, was also decreased in HIF-PPN and post-MI hearts. Subcellular fractionation showed CAMK2γ v1 was found in the nuclear and cytoplasmic fractions, and abundance decreased in both fractions post-MI. Chromatin immunoprecipitation analysis of HIF1 in post-MI hearts also demonstrated direct HIF1 binding to *CAMK2G*. CaMK2 is a key transducer of calcium signals in both physiological and pathological settings. The predominantly expressed isoform in the heart, CaMK2δ, has been extensively studied in cardiac injury, but the specific role of CaMK2γ is not well defined. Our data suggest that loss of CaMK2γ after MI is HIF1-dependent and may play an important role in the heart’s calcium signaling and transcriptional response to hypoxia.

## Introduction

Ischemic heart disease, resulting from occlusion of coronary arteries, is a major cause of morbidity and mortality in the developed world and can manifest as myocardial infarction, sudden cardiac death, and heart failure (ischemic cardiomyopathy)^1^. The often abrupt loss of oxygen delivery produces hypoxia and triggers metabolic and oxidative stress, particularly in an organ with high energy requirements^2^. Subsequently, the heart undergoes a number of adaptive and maladaptive changes in response to hypoxia, which can further impair heart function and ultimately lead to heart failure^3^.

Hypoxia inducible factor 1 (HIF1) is the central actor of an ancient, highly conserved pathway that responds to low oxygen conditions. This transcription factor is composed of two subunits- HIF1β, which is constitutively expressed, and the oxygen-sensitive HIF1α. In normoxic conditions, prolyl and asparaginyl hydroxylases modify residues on HIF1α that limit its transcriptional activity and target the protein for degradation^4^. During hypoxia, these post-translational modifications are limited, which allows HIF1α to enter the nucleus, dimerize with HIF1β, and bind to genomic hypoxia response elements (HREs) to promote transcription^4^. Our lab generated a transgenic model (termed HIF-PPN) in which the three amino acids of HIF1α that are typically hydroxylated (Pro402, Pro564, and Asn803) were mutated to stabilize the protein under normal oxygen conditions^5^. When HIF-PPN was expressed in cardiomyocytes in vivo, we observed a substantial decrease in cardiac contractility and reduced calcium flux, which correlated with decreased expression of excitation–contraction coupling genes including SERCA2, RYR2 and PLN^5^.

Alternative splicing has emerged as an important way to modify and diversify transcripts in order to respond to environmental stimuli. Recently HIF1-dependent changes in splicing factor expression were found to drive the maladaptive fructose metabolism observed in heart disease^6^. Given the crucial role of calcium signaling to proper heart function and the calcium-dependent dysfunction in our HIF-PPN hearts, we examined changes in alternative splicing that could affect calcium handling. We found that calcium/calmodulin dependent protein kinase II, gamma (*CAMK2G*) was alternatively spliced in our HIF-PPN hearts, as well as after coronary artery ligation, which mimics conditions observed during a myocardial infarction^7^.

CaMK2 is an important signaling molecule that is involved in a number of maladaptive responses to cardiac stress^8–12^. There are 4 isoforms of CaMK2 (α, β, δ, and γ), which retain largely identical functional domains and form a 12-subunit holoenzyme that is activated by, and propagates, calcium-dependent signaling cascades^13,14^. In the heart two isoforms of CaMK2, calcium/calmodulin dependent protein kinase II, delta (CaMK2δ) and CaMK2γ, are predominant^15^. CaMK2δ, which is expressed at much higher levels than CaMK2γ, is known to be involved in pathological signaling in numerous models of heart disease^15,16^. Most studies have focused on CaMK2δ, and very little is known regarding CaMK2γ. In this study, we demonstrate that CaMK2γ is substantially decreased after myocardial infarction through alternative splicing, and that this change appears to be at least partially dependent on HIF1.

## Materials and methods

### Mice and reagents

All animal work was performed in accordance with relevant guidelines, including the Guide for the Care and Use of Laboratory Animals, AAALAC and University of Hawaii Animal Welfare Program. All animal protocols and experiments were approved by the Institutional Biosafety Committee and the IACUC of the University of Hawaii at Manoa. All studies were also performed in compliance with the ARRIVE guidelines. HIF1α-PPN/tTA mice were generated as described previously^5^. Briefly, mice with αMHC-driven expression of the tetracycline transactivator (tTA) (Jackson strain 003170), were bred with a mutated HIF1α transgenic line driven by a tetracycline-response element. The mutated HIF1α has 3 amino acid substitutions (Pro402, Pro564 and Asn803 to Ala; denoted HIF-1α-PPN) that allow its stable expression under normal oxygen conditions. The HIF-PPN transgene also includes a carboxy-terminal HA tag to distinguish the transgene product from the endogenous protein. Double transgenic mice (tTA/HIF1α-PPN) were maintained on 200 μg doxycycline per ml of 2.5% sucrose-water to suppress HIF-1α-PPN expression from conception. Doxycycline was removed for 3 days to induce expression of HIF-PPN while control mice continued to receive doxycycline. All experiments used 6 to 8-week old male mice. For myocardial infarction experiments, C57BL/6 male mice between 8–12 weeks of age (> 25 g) were used. When possible, littermate controls were used.

Antibodies used in this study were purchased from the following sources: rabbit anti-Camk2 gamma (Novus Biologicals, NBP2-15686), rabbit anti-histone H3 (Proteintech, 9715S), rabbit anti-mouse HIF1α (Novus Biologicals, NB100-479), mouse anti-rabbit GAPDH (Sigma, clone GAPDH-71.1, G8795 or Thermo Fisher, clone 6C5, AM4300), rabbit anti-human Rbfox1 (Invitrogen PA5-103622), and mouse anti-myc tag (Invitrogen MA1-21316, Myc.A7). The HIF-PPN plasmid was generated using a CMV promoter and human HIF-1α cDNA with the same mutations as described in the HIF-PPN mouse. Recombinant myc-tagged Camk2g isoforms were purchased from Origene and used to validate Camk2 gamma antibody (Supplementary Fig. 1). Rbfox1 antibody data is shown in Supplementary Fig. 2.

### RNA isolation and real-time PCR

To isolate total RNA, frozen heart tissue was pulverized and processed using a Qiagen RNeasy or miRNeasy mini kit according to manufacturer’s instructions. 1 μg RNA was then reverse transcribed using a Quanta qScript cDNA Synthesis Kit. Semi-quantitative real-time PCR was performed using FastStart Universal SYBR Green Master Mix (Roche) or QuantiTect SYBR Green PCR Kit (Qiagen). Primers were designed (Table 1) or purchased from Qiagen (Rbfox1, QuantiTect Primer Assay). When possible, primers were designed to span exon-exon junctions. Samples were run on a 7900HT Fast Real-Time System or QuantStudio 12 K Flex Real-Time PCR System (Applied Biosystems) and relative expression was calculated by ΔΔC_t_ according to standard methods. Tangerin (*EHBP1L1*) transcript was used for normalization as its expression has previously been shown to be unaltered by hypoxia^5^. Expression was then further normalized to the mean value of the control group (“on dox” or “sham I” heart fractions), with data displayed as fraction thereof (“on dox” or “sham I” set to a fold change value of 1.0).

**Table 1:** Primer sequences for real-time PCR.

Gene/transcript	Forward sequence	Reverse sequence
Camk2d (total)	GATGGGGTAAAGGAGTCAACTG	GATGGGGTAAAGGAGTCAACTG
Camk2g (total)	GATGGGGTAAAGGAGTCAACTG	GCAGCATATTCCTGCGTAGATG
Camk2g v1	ACCATGCTTGTCTCCAGGAACTTTT	TTTGTTGTTGCTCTGTGGCATTAGG
Camk2g v2	ACCATGCTTGTCTCCAGGAACTTTT	TTTTTGTTGTTGCTCTGTGGCTTGA
Camk2g v3	ACCATGCTTGTCTCCAGGAACTTTT	GTGGTTTGTGGTTCCTTGACACC
Ehbp1l1 (tangerin)	TTCCAGTTTGTGGCGTGTTAC	TTCCGCCGAGTCCATACCA
Rbfox1	TGACAGTTACGGACGAGTTTATG	GAACGAGACCCACATCATCAG

### RNA sequencing

Total RNA was isolated as described above and sent to the Virginia Bioinformatics Institute at Virginia Polytechnic Institute and State University for preparation, Illumina-based sequencing (paired-end) and analysis. Sequencing files were converted to fastq files and demultiplexed using CASAVA 1.8.0. Data quality was queried using a suite of custom perl and R scripts to calculate the mean quality score at each position read, as well as calculate the relative abundance of each nucleotide. Following quality control, sequences were trimmed for adaptor sequence and were matched to ensure that the same read was present in both the forward and reverse fastq files. Data analysis was performed using the Tuxedo suite of programs, which includes Tophat to map reads against the Bowtie build index of NCBI 37.1 mouse reference genome^17,18^. Cufflinks was used for quantitation and normalization. Cuffcompare/cuffdiff was used to identify differentially expressed genes and splicing events^19,20^. Subsequent analysis was performed using a similar approach with updated tools (Prinseq, Bowtie 2.2.5, Tophat 2.0.14, cufflinks 2.1.1 and Samtools 1.2) and genome build mm10 from UCSC. RNA-seq data from WT hearts 1 day and 3 days post-MI were generated as previously described^7^. Both the differentially expressed genes and alternatively spliced genes with a log_2_ (fold change) ≥ 1 or ≤ -1 (i.e. fold change ≥ 2) and FDR < 0.05 were analyzed by the functional annotation tool in the Database for Annotation, Visualization and Integrated Discovery (DAVID, v.6.8) program to search for enriched gene ontology (GO) terms^21^. All primary RNA-seq data are available on Gene Expression Omnibus under accession number GSE148351 and GSE104187.

### Myocardial infarction

To model myocardial infarction we performed permanent left anterior descending artery (LAD) ligation as previously described^7^. Briefly, the chest was depilated and mice were intubated for anesthesia with isoflurane. After left-lateral thoracotomy between the third and fourth rib, the pericardial sac was opened and LAD was ligated with 7.0 silk. Ischemia was confirmed by ST segment elevation on electrocardiogram (Power Laboratory, ADInstruments) and tissue blanching observed before incisions were sutured. Control sham surgeries were performed identically without artery ligation. At designated time points, mice were euthanized by CO_2_. Hearts were then removed, perfused with PBS, and further dissected into ischemic (free wall and border) and remote fractions, and flash frozen in liquid nitrogen.

### Western blotting

To prepare protein lysates, frozen whole heart tissue was pulverized before incubation with RIPA buffer plus protease inhibitors (complete mini protease inhibitor cocktail tablets, Roche). BCA assays were used to quantify protein concentrations according to manufacturer’s protocols (Pierce). Equal amounts of lysate (10–20 μg) were run on 8% SDS-PAGE gels (Tris–HCl) under reducing conditions before transfer to PVDF membrane (Immobilon-FL, EMD Millipore). Membranes were incubated with Odyssey blocking buffer (LI-COR) and probed with primary antibodies at room temperature for 1 h or 4 °C overnight. All primary antibodies were used at a 1:1000 dilution except GAPDH (Sigma, 1:20,000 or Thermo Fisher, 1:4000). Blots were then washed in PBS + 0.1% Tween-20 before incubation with secondary antibody (IRDye® 800CW Donkey anti-Goat IgG, IRDye® 800CW Donkey anti-Rabbit IgG, or IRDye® 680RD Donkey anti-Mouse IgG) diluted at 1:10,000, for 1 h at room temperature. Blots were imaged using a Li-Cor Odyssey IR Imaging System and quantified by Image Studio densitometry analysis software.

### ChIP semi-quantitative PCR

Preparation of chromatin was performed as previously described^7^. Heart tissue was isolated from naïve and 1 day post-MI mice. After perfusion with PBS, the ischemic (free wall) portion of heart was dissected and weighed before flash freezing in liquid nitrogen. Tissue was pulverized before 30 min incubation with 1% formaldehyde in PBS at room temperature. 1.25 M glycine was added to a final concentration of 0.125 M and incubated for 5 min. Samples were then centrifuged for 200 xg for 10 min at 4 °C, washed and briefly homogenized in cold PBS using a PowerGen 125 homogenizer (Fisher Scientific) at medium power. Samples were centrifuged at 2300 xg for 5 min at 4 °C and resuspended in RIPA buffer containing protease inhibitors. Tissue was then incubated at 4 °C for 1 h before dounce homogenization and sonication using an EpiShear Probe Sonicator (Active Motif) for 20 pulses (30 s on/30 s off). MAGnify Chromatin Immunoprecipitation System (Life Technologies) was then used to isolate and purify DNA according to manufacturer’s instructions. Rabbit IgG and anti-HIF1α antibodies were used for immunoprecipitation. Purified DNA samples were run on a QuantStudio 12 K Flex Real-Time PCR System (Applied Biosystems) using primers listed in Table 2. Percent of total input DNA was then calculated.

**Table 2:** Primer sequences for ChIP PCR.

Gene	Forward sequence	Reverse sequence
Exon 1 HRE	TCTCCTCCTCTTGCTCCCT	TTGCCGAGCTCCTCGAAA
Intron 7 HRE	CTGTCAGCACTGCATTCTT	ACTATATAAGCTTTGACTCAGTACC
Intron 14 HRE	CCAGCGTGCACCTAATG	CACAGACATGCAGTGAGG

### Nuclear fractionation

Subcellular fractionation was performed as described previously^7^. Frozen, pulverized heart tissue was reconstituted in STM buffer and incubated on ice for 30 min. Lysate was vortexed for 15 s before centrifugation at 800 xg for 15 min at 4 °C. Supernatant was collected and used as cytoplasmic fraction. The pellet was resuspended again in STM buffer, vortexed and spun at 800 xg. The remaining pellet was next resuspended in NET buffer, vortexed for 15 s and then incubated on ice for 30 min. Samples were then sonicated using a Fisher Scientific Sonic Dismembrator (Model 100) once for 5-10 s at a power of 5 before centrifugation at 9000 × *g* for 30 min at 4 °C. Supernatant from this spin was collected as nuclear fraction. Protein was quantified by Bradford or BCA assay (Bio-Rad or Pierce, respectively) and equal amounts were loaded on 8% SDS-PAGE gels for western blotting as described.

### Cell culture

The murine HL-1 cell line (atrial cardiomyocyte-derived) was provided by Dr. Claycomb (Louisiana State University Medical Centre), and maintained in Claycomb Media as described^22^. For HIF-PPN experiments, cells were transfected with 500 μg HIF-PPN plasmid for 24 h using Lipofectamine LTX Plus (Thermo Fisher) according to manufacturer’s instructions. For hypoxia induction, cells were exposed to 1% O_2_ for 6–24 h using an InvivO2 400 hypoxia workstation (Baker-Ruskinn). Control cells were kept in a normal oxygen environment (21% O_2_). Cells were then washed with deoxygenated PBS and harvested in RIPA buffer or RNAlater for subsequent analysis.

### Statistics

Data were analyzed using GraphPad software. After assessment of normality, data were assessed using Student’s t-test for parametric and Mann–Whitney for non-parametric testing. For multiple groups, one-way ANOVA with Tukey test for multiple comparisons was used. Error bars indicate standard deviation (SD) unless otherwise indicated. A *p* value less than 0.05 was considered statistically significant.

## Results

### Differential gene expression

**Figure 1:**
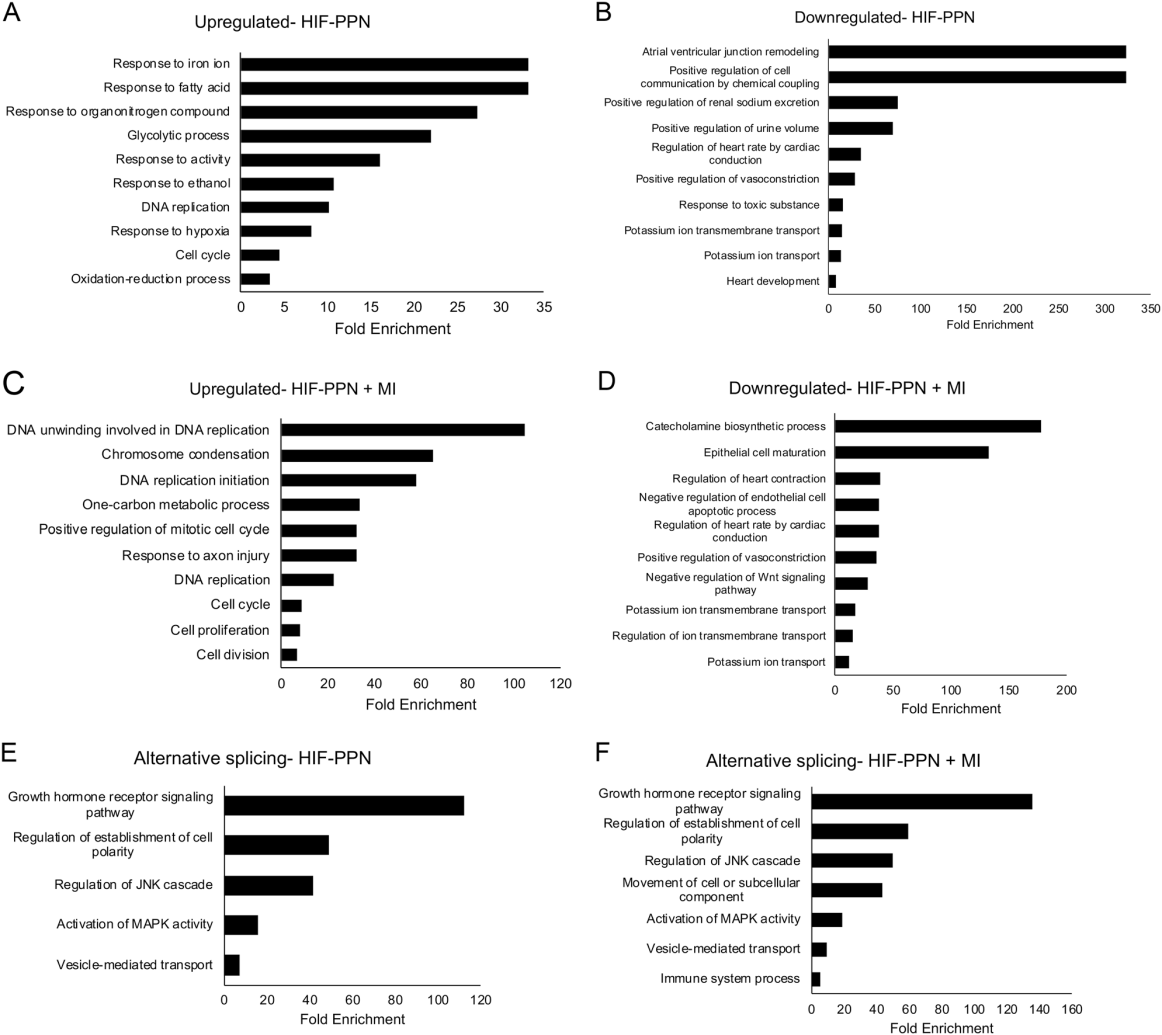
DAVID analysis of differentially expressed genes (DEGs) in HIF-PPN hearts. (**A**,**B**) Both up- (**A**) and downregulated DEGs (**B**) by log_2_ (fold change) ≥ 1 or ≤  − 1 3 days after HIF-PPN induction were analyzed by DAVID to find most dysregulated pathways using gene ontology (GO) terms for biological process. (**C**,**D**) GO enrichment for genes upregulated (**C**) and downregulated (**D**) by at least log_2_ (fold change) ≥ 1 or ≤  − 1 in both HIF-PPN and post-MI hearts. (**E**,**F**) Enriched GO terms for alternatively spliced genes in HIF-PPN hearts (**E**) and HIF-PPN plus post-MI hearts (**F**) were analyzed using the same method. Bar graphs show top GO terms as ranked by *P*-value (or EASE score, determined by modified exact Fisher’s test) as well as fold enrichment for each term. All *P* < 0.05.

To gain a comprehensive view of HIF-driven transcriptional changes, we performed RNA sequencing on HIF-PPN hearts (n = 3 per group). Using a log_2_ fold change ≥ 1 and FDR < 0.05 as a cutoff, we found a total of 131 genes upregulated after 3 days of induction (by removal of doxycycline water) while 78 genes were downregulated compared to the control doxycycline group (Supplementary Table 1). Gene ontology (GO) analysis indicated upregulated genes were largely associated with metabolism and proliferation, while downregulated genes were involved in cell communication and ion transport, affecting cardiac conduction and function (Fig. 1A,B).

While our HIF transgenic mice provide a useful model to examine HIF1-driven biology, the supra-physiologic levels of HIF expressed in these mice under normal oxygen conditions may not robustly model clinically relevant scenarios. We previously examined the initial transcriptional response after MI, a timeframe in which HIF1 is active^7^. We therefore compared differentially expressed genes in HIF-PPN hearts to those found after MI to determine which genes were similarly expressed in a more pathologically relevant mouse model. Using the same cutoff parameters as above, we found 54 of 131 genes in HIF-PPN hearts were also upregulated at 1 day and/or 3 days post-MI, while 45 of 78 were downregulated (Supplementary Tables 2–3). Unsurprisingly, upregulated genes were associated with processes known to have HIF1 involvement such as DNA replication and cell proliferation (Fig. 1C)^23^. Downregulated genes were associated with cell maturation, cardiac conduction and ion transport (Fig. 1D).

### Alternative splicing in HIF-PPN hearts

We were also interested in alternative splicing events that could be HIF1 mediated, especially those involved in calcium handling. We detected 52 candidate genes with substantial splicing changes after HIF induction through informatics analyses (Supplementary Table 4). Alternative splicing was also observed in 41 of these genes after MI (Supplementary Table 5). These genes were generally associated with cell signaling (MAPK, JNK) and movement (vesicles, cell polarity, microtubules) (Fig. 1E,F). Given the variation in splicing prediction software results, we examined this list to identify a relevant gene for further validation. Interestingly, based on the informatics results, many of the expression patterns for their individual transcripts appeared to be different in HIF-PPN versus ischemic hearts. However, one alternatively spliced gene of particular interest did have agreement between the HIF-PPN and MI transcript expression patterns—calcium/calmodulin dependent protein kinase II gamma (*CAMK2G*). Although aberrant CaMK2 signaling has long been associated with cardiac pathologies, the specific role of CaMK2γ in the heart is not well understood. Even less is known about *CAMK2G* splicing, so we examined its expression in more detail.

### CAMK2G splicing altered in HIF-PPN and post-MI hearts

**Figure 2:**
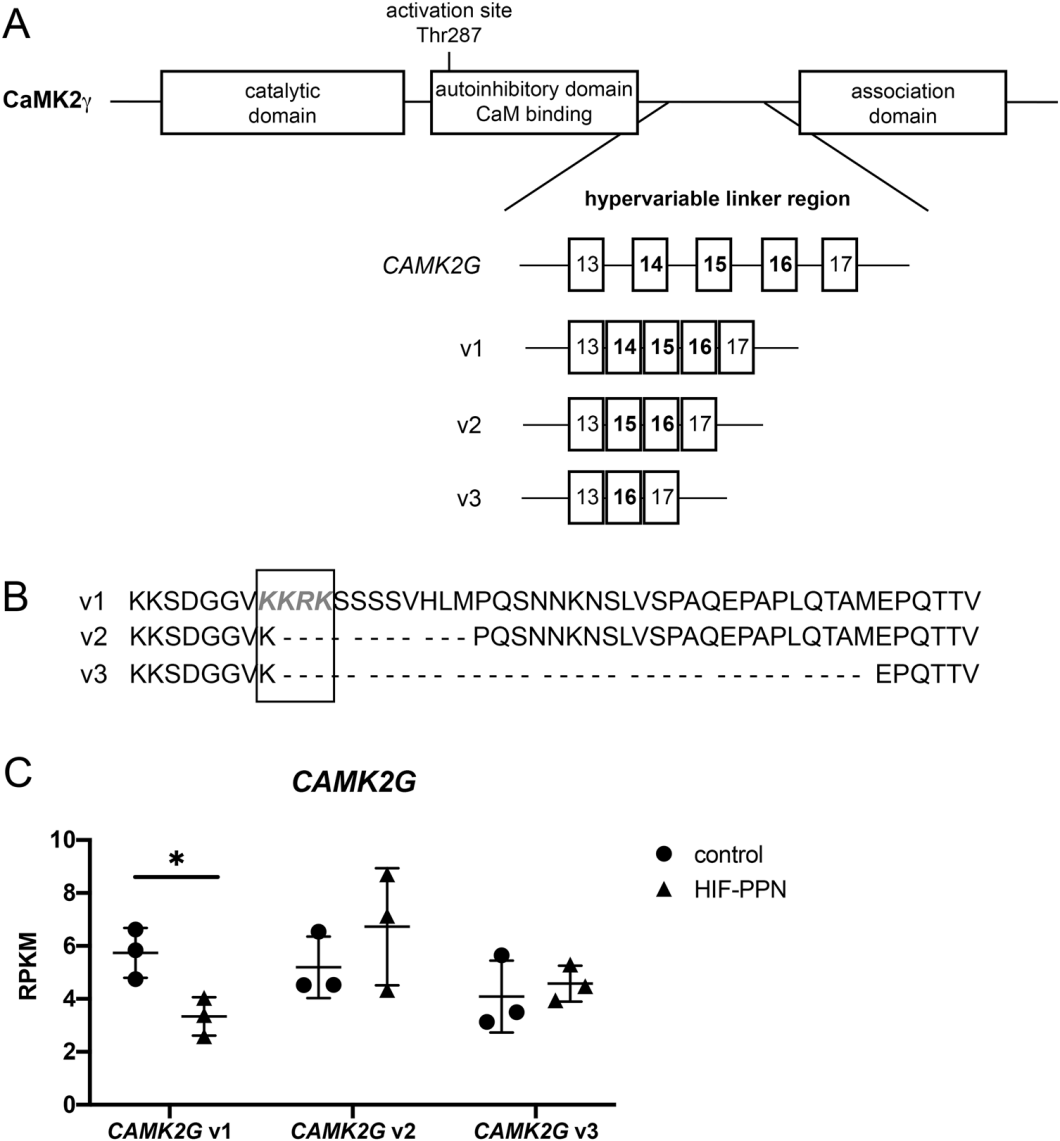
*CAMK2G* splicing is altered in HIF-PPN hearts. (**A**) Schematic depicting CAMK2γ functional domains and its splice variants. The isoforms differ within the hypervariable linker region, with variant 1 (v1) retaining all exons. Variants 2 (v2) and 3 (v3) lack exon 14 or exons 14–15, respectively. (**B**) Amino acid sequences for CaMK2γ isoforms within the hypervariable region. Variant 1 (v1) is the longest isoform and retains a nuclear localization sequence (KKRK, highlighted in gray and outlined by black box) that is spliced out of the other two isoforms. (**C**) Expression of *CAMK2G* splice variant RNA 3 days after HIF-PPN induction as assessed by RNA sequencing. Data displayed as reads per kilobase of exon per million reads mapped (RPKM) and compared to control mice (on dox). n = 3 per group. Student’s *t* test. *p = 0.03.

Our RNA-seq analysis identified three splice variants of *CAMK2G*, all of which share homology within each of the functional domains. These transcripts differ solely in the hypervariable region. Variant 1 (v1) retains all exons while exon skipping produces variants 2 (v2) and 3 (v3) that lack exon 14 or exons 14–15, respectively (Fig. 2A). Of note, v1 contains a nuclear localization sequence (KKRK) that is lost in the other two splice variants (Fig. 2B). Interestingly, when we examined the expression changes in *CAMK2G* isoforms by RNA we found v1 was dramatically downregulated in HIF-PPN hearts while v2 and v3 were slightly upregulated (Fig. 2C).

**Figure 3:**
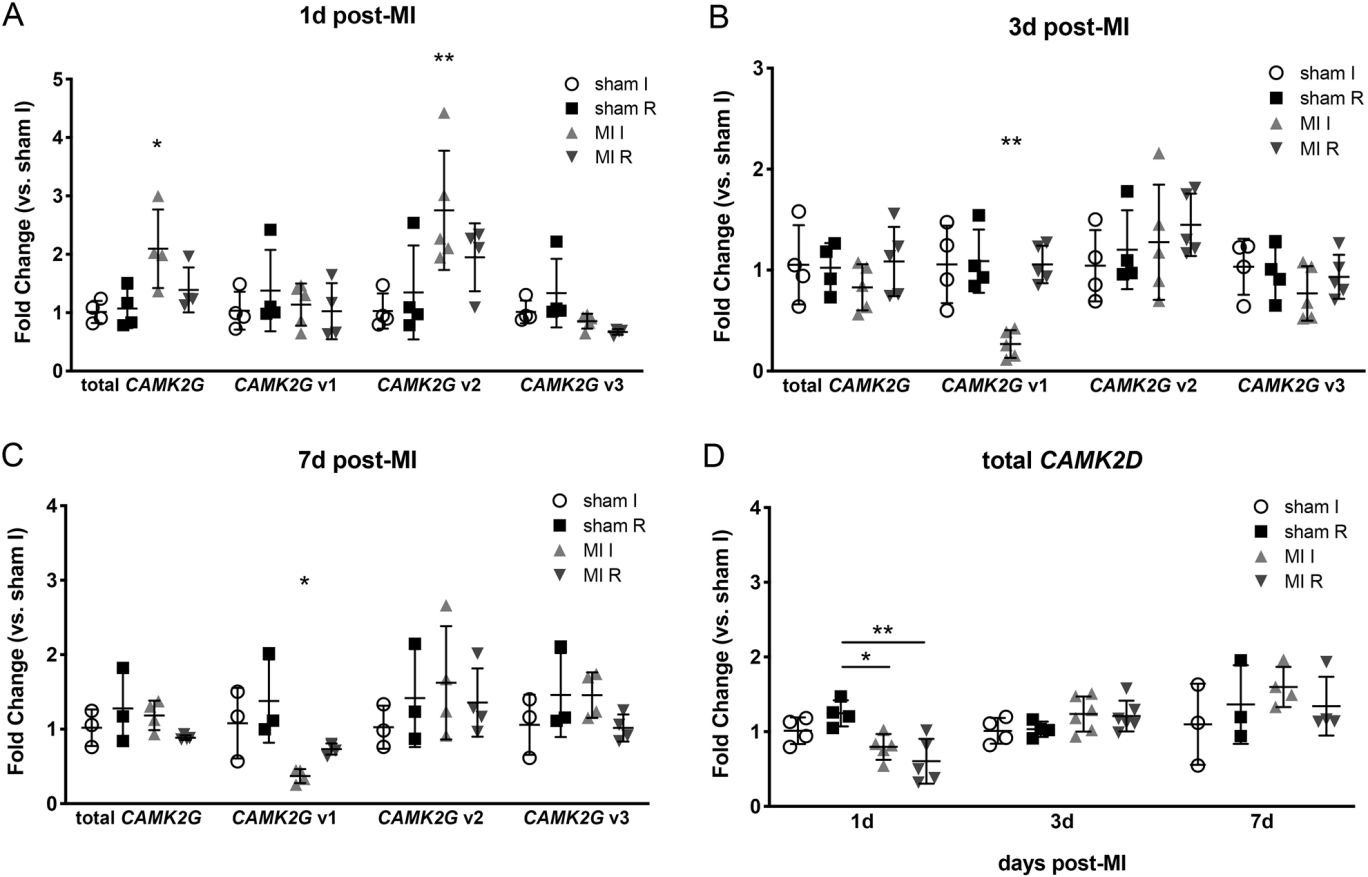
Real-time PCR reveals decrease in *CAMK2G* v1 expression post-MI. (**A**–**C**) rt-PCR data for total *CAMK2G* and splice variant expression in WT hearts at 1 day (**A**), 3 days (**B**) and 7 days post-MI (**C**). Using one-way ANOVA with Tukey’s multiple comparisons, overall *p* values for total *CAMK2G*, *CAMK2G* v1, *CAMK2G* v2 and *CAMK2G* v3, respectively, were *p* = 0.015, *p* = 0.47, *p* = 0.021 and *p* = 0.05 for panel A, *p* = 0.57, *p* = 0.0005, *p* = 0.56 and *p* = 0.48 for panel B and *p* = 0.28, *p* = 0.014, *p* = 0.63 and *p* = 0.26 for panel C. (**D**) Total *CAMK2D* expression over the course of 7 days post-MI as assessed by rt-PCR. Using one-way ANOVA with Tukey’s multiple comparisons, overall *p*-values were *p* = 0.0037, *p* = 0.20 and *p* = 0.53 for 1 day, 3 days and 7 days post-MI, respectively. *p < 0.05, **p < 0.01 for between group comparisons. I = ischemic tissue (infarct + border), R = remote. Data displayed as fold change compared to sham I fraction. n = 3–6 for each time point. *p*-values are for MI I vs. all other groups unless otherwise indicated.

We then examined *CAMK2G* splice variant expression in our pathological model of MI in WT mice. Real-time PCR data revealed that while total RNA levels for *CAMK2G* were largely unchanged over the first week after MI, v1 was significantly decreased in infarcted tissue by 3 days after MI and remained low at 7 days (Fig. 3A–C). In contrast, while v2 showed a small increase 1 day after MI, v2 and v3 expression remained relatively constant even after injury (Fig. 3A–C). Since *CAMK2D* is the predominately expressed isoform in the heart, we wanted to examine any changes in its overall expression for comparison. We observed a decrease in *CAMK2D* RNA in ischemic tissue 1 day after MI, but this decrement was no longer seen by 3 days post-MI (Fig. 3D).

### CaMK2γ v1 decreased after MI

**Figure 4:**
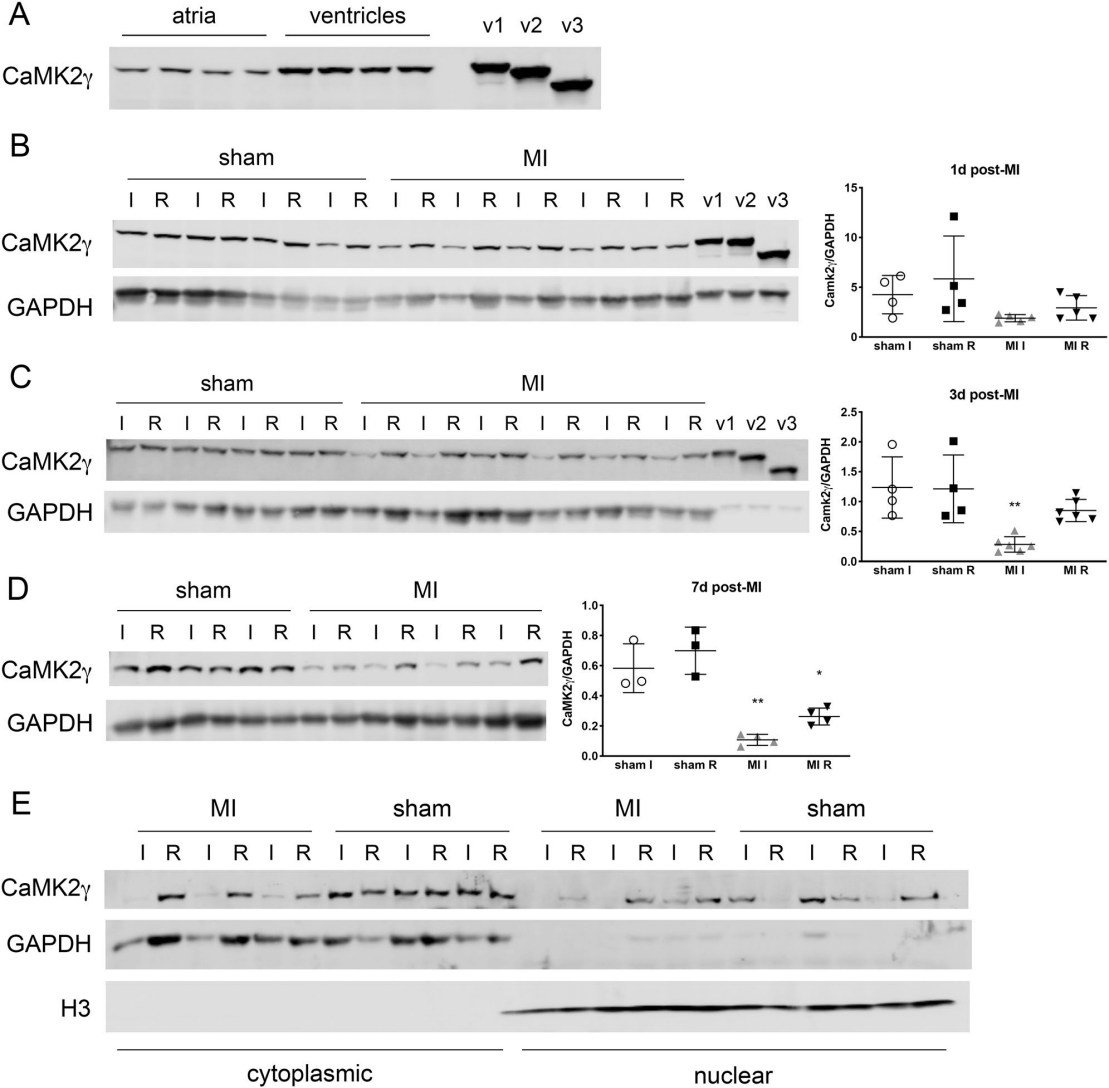
Sustained loss of CaMK2γ v1 expression in WT hearts 1 week post-MI. (**A**) CaMK2γ variant protein expression in naïve WT atria and ventricles. n = 4 mice. Recombinant proteins for each CaMK2γ variant were used to confirm identities of each isoform. (**B**–**D**) Western blotting for CaMK2γ splice variants in WT hearts at 1 day (**B**), 3 days (**C**) and 7 days post-MI (**D**). n = 3–6 for each time point. Cropped blots shown, with GAPDH used for loading control and normalization, with quantification graphs to the right of the blots. For B–D, overall *p*-values were *p* = 0.10, *p* = 0.0017 and *p* < 0.0001, respectively, using one-way ANOVA with Tukey’s multiple comparisons. *p < 0.05, **p < 0.01 for between group comparisons. *p*-values are for MI I (or MI R) vs. all other groups. (**E**) Western blots of CaMK2γ expression in cytoplasmic and nuclear fractions of WT heart tissue 3 days post-MI. GAPDH denotes cytoplasmic portion while histone H3 was used to identify the nuclear fraction. n = 3 per group. I = ischemic tissue, R = remote. Uncropped western blots shown in Supplementary Fig. 3.

We next wanted to determine how these changes in RNA would ultimately affect protein expression. We generated recombinant versions of each splice variant with a C-terminal myc tag to serve as positive controls and confirmed their expression using both CaMK2γ and myc-specific antibodies (Supplementary Fig. 1). While the myc tag meant the recombinant protein was slightly larger than its physiological counterpart (~ 1.2 kDa), it was a small enough difference in size to still allow unambiguous identification of each splice variant by western blotting. Curiously, we found only v1 was expressed as protein in both WT atrial and ventricular tissue (Fig. 4A). This remained true after MI, with no appearance of either v2 or v3 by western blot up to 1 week after MI (Fig. 4B–D). Similar to v1 RNA, v1 protein was quickly and dramatically decreased in infarcted tissue after MI compared to sham hearts, a decrease that was sustained up to 7 days after injury (Fig. 4B–D).

Given the nuclear localization sequence in v1, we used subcellular fractionation to assess its localization in the heart 3 days after injury. In sham-operated hearts we found v1 of CaMK2γ in both the nuclear and cytosolic fractions, though more appeared to be retained in the cytoplasm (Fig. 4E). Although v1 was decreased in ischemic tissue after MI, it could still be detected in both fractions of hearts that still had CaMK2γ protein.

**Figure 5:**
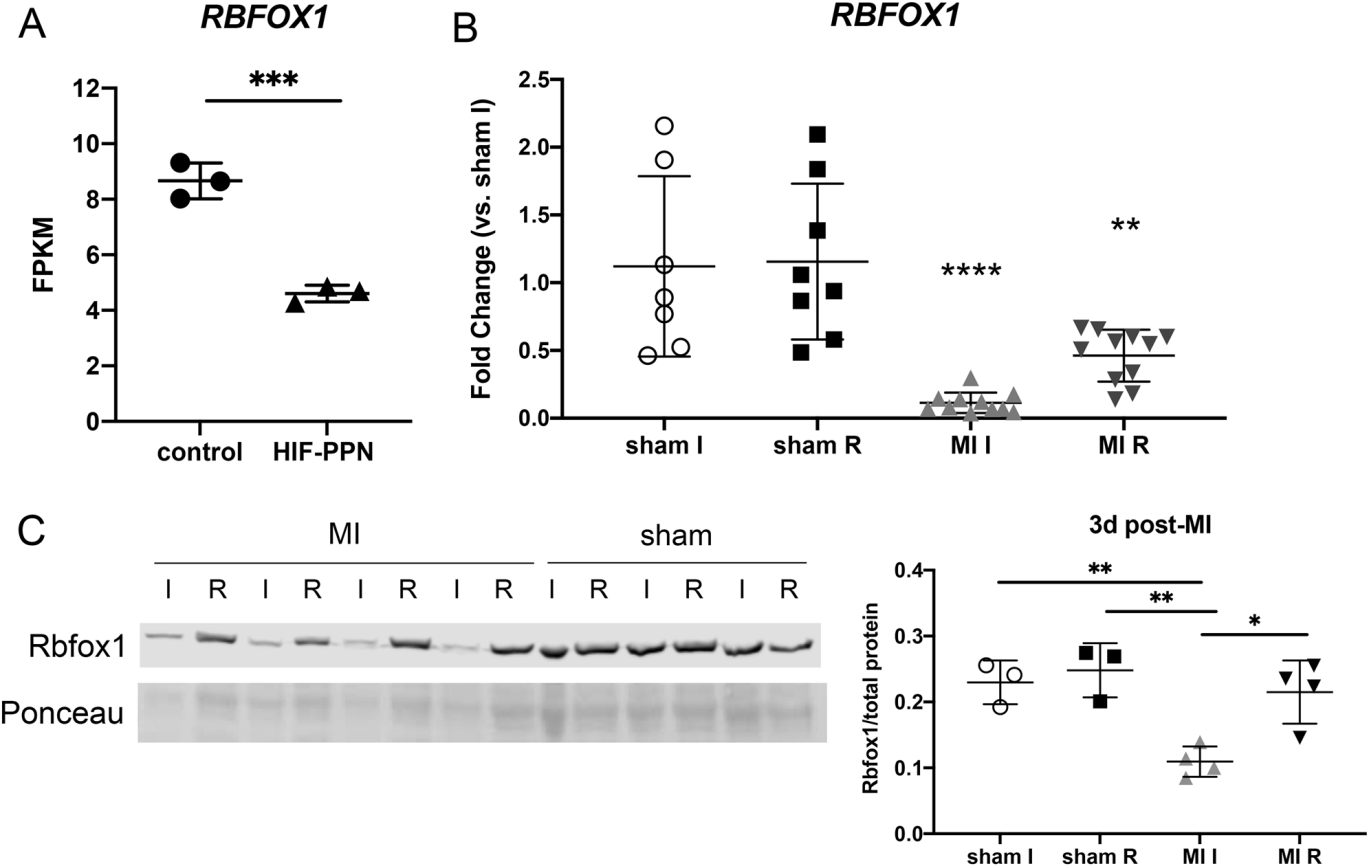
Rbfox1 expression in HIF-PPN and post-MI hearts. (**A**) RNA-seq data for *RBFOX1* RNA abundance in HIF-PPN vs. control (on dox) hearts. n = 3 per group. Student’s *t* test. ***p < 0.001. Data displayed as fragments per kilobase of exon per million reads mapped (FPKM). (**B**) rt-PCR data for *RBFOX1* expression in WT hearts 3 days post-MI. Using one-way ANOVA with Tukey’s multiple comparisons, overall *p*-value < 0.0001. **p < 0.01, ****p < 0.0001 for between group comparisons. n = 7–11 per group. (**C**) Western blot for Rbfox1 in 3d post-MI hearts. Cropped blots shown, with Ponceau staining used to visualize total protein and normalization. Quantification of blot below. For sham n = 3, MI n = 4. Overall *p*-value was *p* = 0.0022 using one-way ANOVA with Tukey’s multiple comparisons. *p < 0.05, **p < 0.01 for between group comparisons. I = ischemic tissue, R = remote. Uncropped western blots shown in Supplementary Fig. 2.

The splicing factor Rbfox1 has been shown to directly bind to *CAMK2G* and modify its splicing in several tissues^24–26^. We therefore examined Rbfox1 expression in HIF-PPN and post-MI hearts to explore its potential role in modulating *CAMK2G* splicing in the heart. Abundance of *RBFOX1* RNA was substantially decreased in both HIF-PPN and post-MI hearts (Fig. 5A,B). Rbfox1 protein was also substantially lower in infarcted tissue 3 days after MI (Fig. 5C).

### HIF1 and hypoxia directly modify CAMK2G splicing

**Figure 6:**
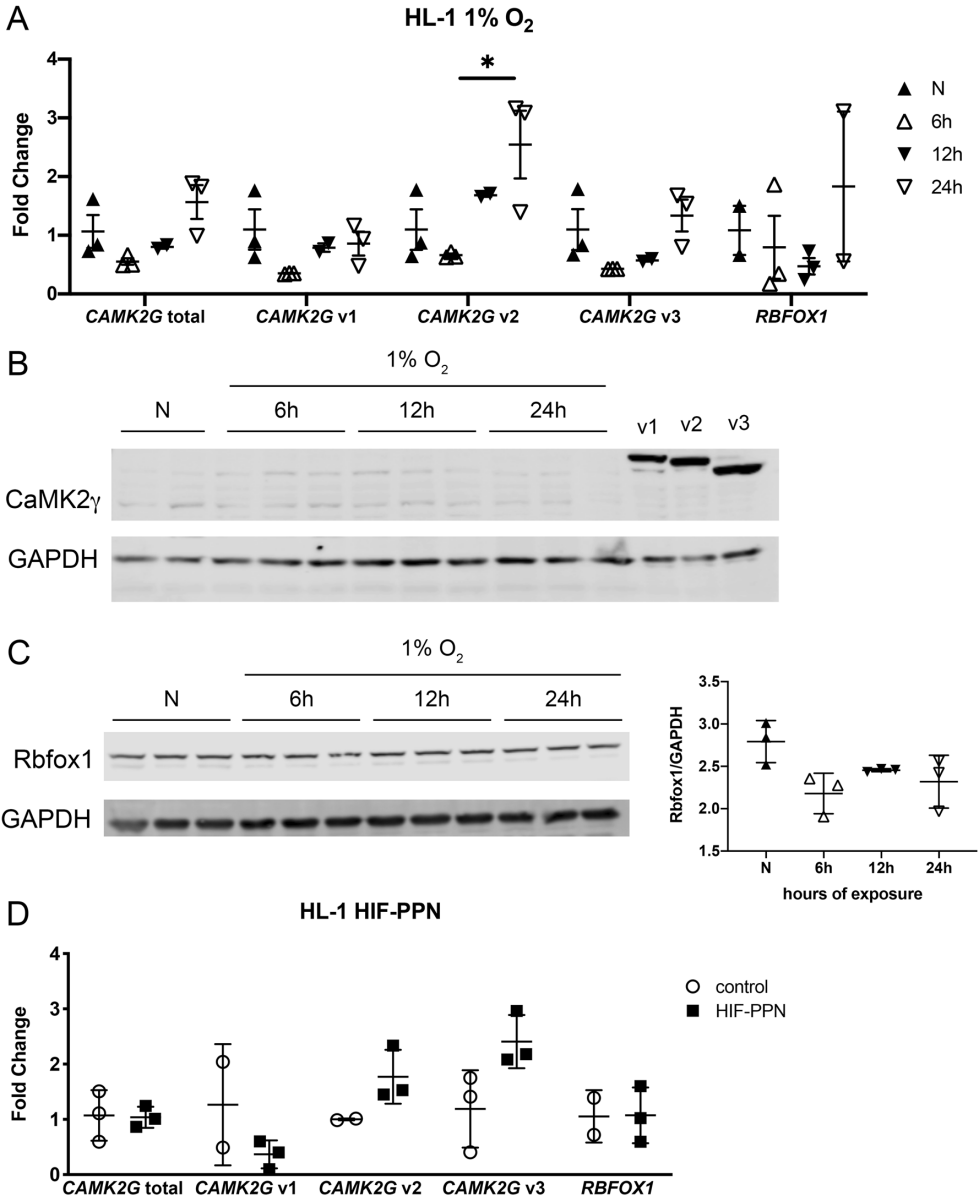
*CAMK2G* splicing with HIF-PPN or hypoxia in vitro. (**A**) rt-PCR data for *RBFOX1* and *CAMK2G* splice variant expression in HL-1 cells exposed to 1% O_2_. Fold change compared to normoxia, n = 2–3 per condition. Overall *p*-values for total *CAMK2G*, *CAMK2G* v1, *CAMK2G* v2, *CAMK2G* v3 and *RBFOX1* were *p* = 0.06, *p* = 0.19, *p* = 0.04, *p* = 0.10 and *p* = *p* = 0.51, respectively, using one-way ANOVA with Tukey’s multiple comparisons. *p < 0.05 for between group comparisons. (**B**,**C**) Western blots for CaMK2γ (**B**) and Rbfox1 (**C**) for HL-1 cells exposed to 1% O_2_. Cropped blots shown, with GAPDH used for loading control and normalization. Recombinant proteins for each CaMK2γ variant were used for comparison. n = 2–3 per condition. For panel D, quantification graph shown to the right. Using one-way ANOVA with Tukey’s multiple comparisons, overall *p*-value = 0.06. Uncropped western blots shown in Supplementary Fig. 4. Data representative of at least 3 independent experiments. (**D**) rt-PCR data for *RBFOX1* and *CAMK2G* splice variant expression in HL-1 cells transfected with HIF-PPN. Fold change compared to control, n = 2–3 per group. Using student’s *t* test, *p* = 0.91, *p* = 0.24, *p* = 0.12, *p* = 0.07 and *p* = 0.97 for total *CAMK2G*, *CAMK2G* v1, *CAMK2G* v2, *CAMK2G* v3 and *RBFOX1*, respectively.

To determine if hypoxia, or HIF1 specifically, could directly regulate cardiac *CAMK2G* splicing, we utilized the atrial-derived cardiomyocyte HL-1 cell line. After exposure to hypoxia, RNA abundance of *CAMK2G* splice variants and *RBFOX1* were similar to those observed in mice, with decreased *CAMK2G* v1 and *RBFOX1* observed within 6 h (Fig. 6A). Although CaMK2γ protein did not appear to be highly expressed by western blotting, Rbfox1 was visible and followed the trend of its RNA expression, with lower expression upon hypoxia exposure (Fig. 6B,C). When HL-1 cells were transfected with HIF-PPN alone, *CAMK2G* splicing clearly mirrored results observed in vivo, with a decrease in v1 and increase in v2 and v3, although this did not reach statistical significance (Fig. 6D). Although *RBFOX1* RNA decreased in HIF-PPN hearts in vivo, its expression did not appreciably change with the addition of HIF-PPN under these conditions in vitro.

**Figure 7:**
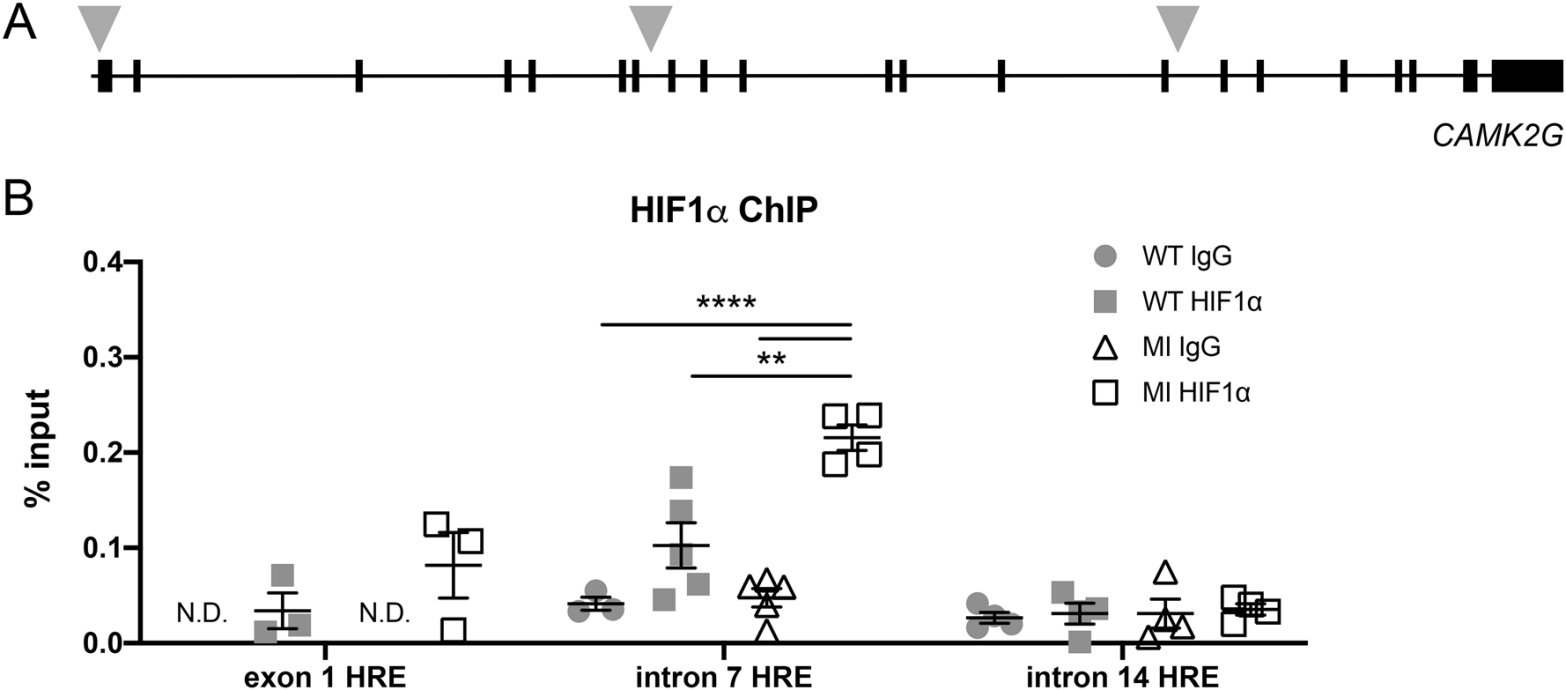
ChIP analysis of HIF1 binding to *CAMK2G*. (**A**) Schematic of *CAMK2G*. Gray arrows indicate potential HIF1 binding sites containing hypoxia response elements (HREs). (**B**) Semi-quantitative PCR analysis of HIF1α enrichment in naïve wild type (WT) and ischemic heart tissue 1 day after MI (MI) by ChIP. HIF1α showed increased binding compared to both WT and IgG controls at the intron 7 HRE. n = 3–5 for each group. Data shown as % input DNA with mean ± SD. Overall *p*-values were *p* = 0.52, *p* < 0.0001 and *p* = 0.95, for exon 1 HRE, intron 7 HRE and exon 14 HRE, respectively, using one-way ANOVA with Tukey’s multiple comparisons. **p < 0.01, ****p < 0.0001 for between group comparisons. N.D. = not detected.

Although HIF1 is typically associated with increased transcription, we examined HIF1 binding sites to determine if it could directly interact with the *CAMK2G* or *RBFOX1* gene to influence its alternative splicing. We could not identify any HIF1 sites for *RBFOX1* but found 3 potential HIF1 sites within *CAMK2G* using rVISTA prediction (exon 1 HRE and intron 14 HRE) and previously reported HIF1 ChIP-seq data from mouse embryos (intron 7 HRE^27^,) (Fig. 7A). ChIP-PCR revealed that only the HRE within intron 7 was significantly enriched for HIF1 binding in ischemic tissue 1 day after MI (Fig. 7B).

## Discussion

HIF1 is an important regulator of the transcriptional response to hypoxic stress. While its role as a transcription factor has been appreciated for some time, its activity can also influence additional layers of gene regulation including splicing and translation^6,7,28^. Previously we found HIF1 overexpression can cause dramatic loss of cardiac contractility, decreased excitation–contraction gene expression, and reduced calcium flux^5^. In this study, we demonstrate HIF1 may also alter calcium signaling through alternative splicing and decreased expression of CaMK2γ.

It is not yet clear how HIF1 controls these splicing changes in *CAMK2G*, but there are several possibilities. First, HIF1 may influence *CAMK2G* splicing by regulating expression of RNA splicing factors. HIF1 can direct alternative splicing of KHK through upregulation of the SF3B1 splicing factor, leading to pathological fructose metabolism in the heart^6^. Rbfox1 can directly modify *CAMK2G* splicing in multiple tissues^24–26^. Deletion of *RBFOX1* has been shown to increase exon skipping in *CAMK2G* splicing in skeletal muscle, and to exacerbate cardiomyocyte hypertrophy in a pressure-overload model^24,26^. Although we did not observe any changes in *RBFOX1* expression with HIF-PPN under our experimental conditions in vitro or identify any potential HIF1 binding sites, its expression was decreased upon exposure to hypoxia, and data from our HIF-PPN and post-MI hearts indicate Rbfox1 is substantially decreased in both conditions (Figs. 5, 6). We also observed a decrease in *CAMK2G* v1 expression and increase in *CAMK2G* v2 and v3 in both in vitro and in vivo models, which would be expected with increased exon skipping from loss of Rbfox1. Based on these results, we deduce that HIF1 can directly regulate *RBFOX1* expression, but we cannot exclude the possibility that other factors are involved. For example, HIF1 binding has also been found at the promoter of serine and arginine rich splicing factor 1 (SRSF1)^27^, and SRSF1 and SRSF2 can regulate splicing of the closely related *CAMK2D* gene in coordination with Rbfox1^29^. We also observed decreased *RBFOX1* expression and increased *SRSF1* and *SRSF2* by RNA-seq in our HIF-PPN hearts (data not shown). These conditions have previously been shown to lead to increased exon skipping and therefore may also help explain the altered *CAMK2G* splicing in our model.

HIF1 could also modify RNA splicing directly. Our ChIP-PCR data show HIF1 is enriched at the intron 7 HRE of *CAMK2G* after MI (Fig. 5). This intron lies between exons within the catalytic domain, so it is not evident how this would influence alternative splicing of *CAMK2G*. However, this site was also enriched in embryonic heart tissue^27^, another hypoxic environment, suggesting this binding may have biological significance. Interestingly, we could not find any potential HIF1 binding sites within *RBFOX1*, so by this limited criterion it does not appear that HIF1 directly regulates its expression. Another possibility is that HIF1 may indirectly regulate splicing through interactions with other splicing factors. Rbfox1 has been shown to bind *CAMK2G* within intron 14^24^, one of the sites for alternative splicing. Therefore, while we could not show direct HIF1 binding at this site, it may work in concert with Rbfox1 or others to regulate splicing.

Alternative splicing of the CaMK2 genes is complicated. Three main variants of *CAMK2G* have been described to date and were the focus of this study. Previous work and recent updates to annotation of *CAMK2G* transcripts indicate there may be a fourth variant^30^, but databases differ on the exact sequence, and we have not evaluated it here. Each of the other potential variants are very similar to the variants we studied, with only minor sequence changes in the hypervariable region. We also only observed one CaMK2γ protein at a similar size to CaMK2γ v1 (Fig. 4). Therefore, even if these newer transcripts are found to be expressed in the heart, it seems unlikely that this would dramatically alter our findings.

There are also several splice variants of *CAMK2D* and some have been demonstrated to have specific cytoplasmic or nuclear activities. Much of the detailed characterization of these variants has been performed on rat transcripts^31,32^. The murine splice variants are more numerous, with varying nomenclature, and have not been a focus of rodent studies^33,34^. Additional differences are seen in alternative splicing of both *CAMK2D* and *CAMK2G* in humans^13,35,36^. *CAMK2G* splicing is substantially more complex than in mice, with 11 protein variants identified thus far and approximately 40 different potential transcripts. Despite the additional complexity, these variations are generally contained within the variable region, as observed in mice. Several isoforms also retain the KKRK nuclear localization sequence and show high homology to the mouse variants studied here, suggesting they may play a similar role in the human heart. Further study will be needed to define the effects of each isoform and its splice variants on human cardiac function.

Ultimately, the most important question is what effects the abundance of these splice variants have on CaMK2γ activity and heart function. While very little work has focused on the physiological role of cardiac CAMK2γ, GWAS and genetic studies in humans have reported SNPs associated with *CAMK2G* in psoriasis, inflammatory bowel disease and neurological diseases^37–41^. Other clinical studies have linked *CAMK2G* to coronary artery disease, altered calcium signaling and heart failure^42–44^, underscoring its role in cardiac disease as well. Characterization of *CAMK2G*^*−*/−^ mice revealed a role in female fertility through modulation of calcium-dependent egg activation^45^, but only a few studies have specifically examined CaMK2γ in the murine heart, where its role was largely defined as complementary substitution for loss of CaMK2δ activity^16,46^. However, work from other systems offer additional insight into its potential function. Biochemical and structural analysis of CaMK2 suggested the length of the linker (or hypervariable) region could affect the folding of the protein, which would in turn affect assembly of the holoenzyme and ease of autoactivation^47^. Alternative splicing of CaMK2γ could therefore affect its kinase activity. Since we only observed one CaMK2γ protein in heart tissue both before and after injury (variant 1), it seems unlikely that changes in linker length from splicing changes are likely to modulate kinase activity in the heart.

Another hypothesis is that the presence of the nuclear localization sequence (in variant 1) suggests CaMK2γ has nuclear activity. In neurons, it has been shown that, independent of its kinase activity, CaMK2γ served as a calmodulin shuttle from the cytoplasm to the nucleus to activate downstream CaMK-dependent signaling pathways and transcription^48^. Work from our lab and others have shown that HIF1 dramatically decreases cardiac contractility and calcium flux at least partly through reduced expression of SERCA2 and other excitation–contraction coupling genes^5,49^. CaMK2 is thought to propagate and translate short-term changes in calcium transients into a long-term transcriptional and cellular response to environmental changes. It is therefore possible that HIF1 could further alter calcium signaling, and establish longer term transcriptional changes, by altering CaMK2γ splicing and suppressing its nuclear activity.

*CAMK2D* has also been described to have splice variants with varying subcellular localization (e.g. nuclear δ_B_ versus cytoplasmic δ_c_^31,33^). A number of studies have examined the nuclear activity of CaMK2δ_B_ in heart disease and suggest it can activate a hypertrophic gene expression program in response to stress^33,50,51^. It is important to note that the mouse models employed in these studies used a transgene derived from rat cDNA^31,32^, and an equivalent RNA transcript has not yet been fully characterized in the mouse. Establishing the specific role of CaMK2γ is further complicated by work in vitro that suggests monomers of different CaMK2 isoforms and splice variants can be incorporated into the functional holoenzyme^31,52^, although this has not been studied in detail in vivo. Additionally, many studies use a generic CaMK2 antibody that cannot distinguish between the different isoforms, making it difficult to assign specific functions to individual isoforms without proper controls^8,10–12^. It is therefore possible that many of the functions currently attributed to CaMK2δ in the heart may be performed by CaMK2γ, or a holoenzyme composed of different splice variants from both isoforms.

In summary, we have found that HIF1 is involved in a number of transcriptional changes in the heart after ischemic injury, including alternative splicing. In addition to our previously described HIF-dependent alteration of calcium handling through SERCA2, we have now also demonstrated that alternative splicing of CaMK2γ is also modified by HIF1 after infarction. These results suggest a complex interplay between hypoxia and calcium signaling that is likely to influence cardiac response to injury.

## Supplementary Information

Supplementary Information 1.

Supplementary Information 2.

## Data Availability

All primary RNA-seq data are available on Gene Expression Omnibus under accession number GSE148351 and GSE104187. All other data generated or analyzed during this study are included in this published article (and its supplementary files).
