# Supplementary material for: Ischemic heart injury leads to HIF1-dependent differential splicing of CaMK2γ
Source: Sci Rep. 2021 Jun 23;11:13116. doi: 10.1038/s41598-021-92426-2 (PMC8222303; doi:10.1038/s41598-021-92426-2)

Ischemic heart injury leads to HIF1-dependent differential splicing of CaMK2γ

Allison Lesher Williams[1], Chad Walton[1], Blake Pinell[1], Vedbar S. Khadka[2], Brandyn Dunn[1], Katie Lee[1], Ma C.T. Anagaran[1], Abigail Avelar[1] and Ralph V. Shohet[1]

[1]Center for Cardiovascular Research and [2]Bioinformatics Core, John A. Burns School of Medicine, University of Hawaii, Honolulu, HI

Supplementary Information

Supplementary Figures 1-4

Supplementary Tables 1-5 (provided separately in Microsoft Excel file)

Supplementary Figure 1. Validation of CaMK2γ splice variant plasmids and Camk2γ antibody. Western blots show expression of recombinant myc-tagged CaMK2γ splice variant proteins in HEK293 cells. (A) Although some non-specific bands are detected by the CaMK2γ antibody (green), the recombinant proteins are clearly seen at the expected moecular weights (~56, 54 and 52 kDa for variants 1, 2 and 3, respectively). GAPDH (red) used for standardization. Lanes 1-3 = non-transfected cell lysate, lanes 4-6 = CaMK2γ v1, lanes 7-9 = CaMK2γ v2, lanes 10-12 = CaMK2γ v3. (B) Same samples from panel A were probed with anti-myc tag antibody (red) and were able to detect the recombinant proteins at the same sizes as those observed with the CaMK2γ antibody.

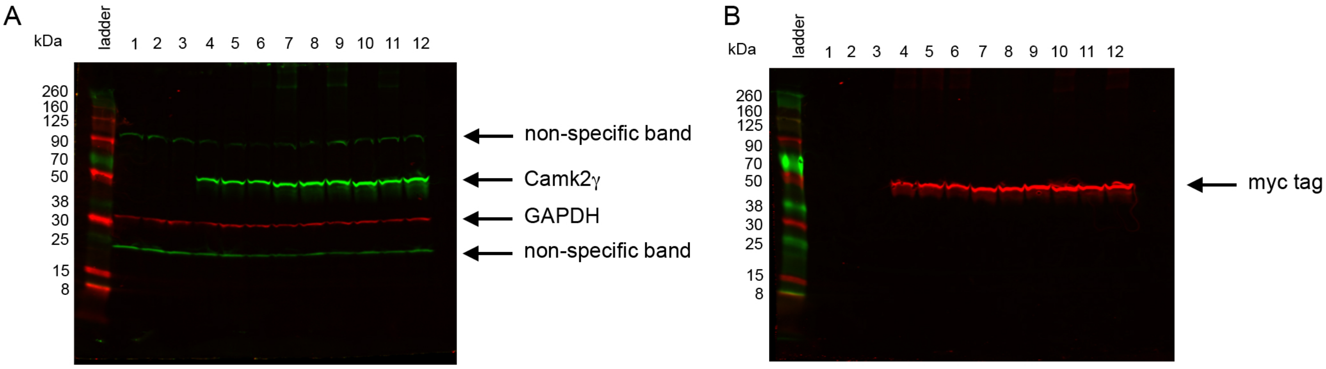

Supplementary Figure 2. Rbfox1 antibody validation and uncropped western blots from Figure 5. Western blot for Rbfox1 (left) and Ponceau staining (right). Rbfox1 antibody only recognizes a single band at the expected molecular weight (~45 kDa).

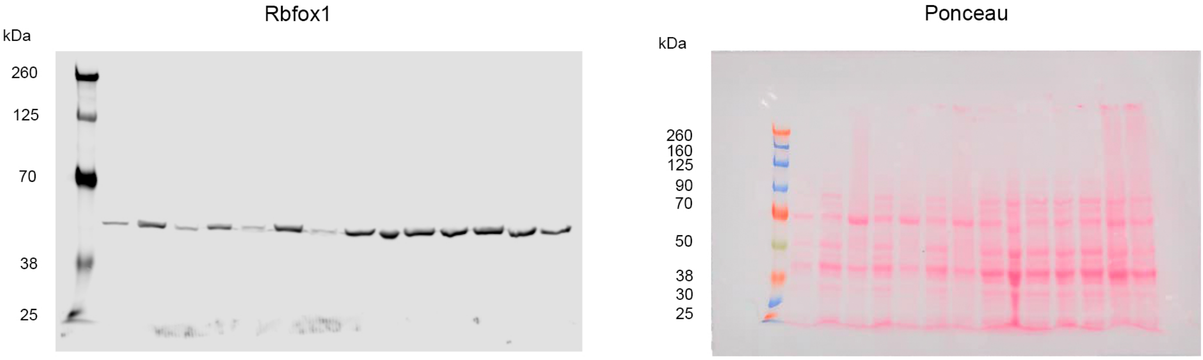

Supplementary Figure 3. Uncropped western blots from Figure 4. Panels correspond to the same panels as in Figure 4. CaMK2γ blots on left and GAPDH blots on right. Panel D also includes staining for SERCA2 (left) and β-actin (right), and Panel E includes staining for histone H3 (indicated by arrows). NB: Blots in Figure 4D only shows lanes 1-14 after the ladder. Uncropped blots also show lanes 15-20, which include samples that were not relevant for this study.

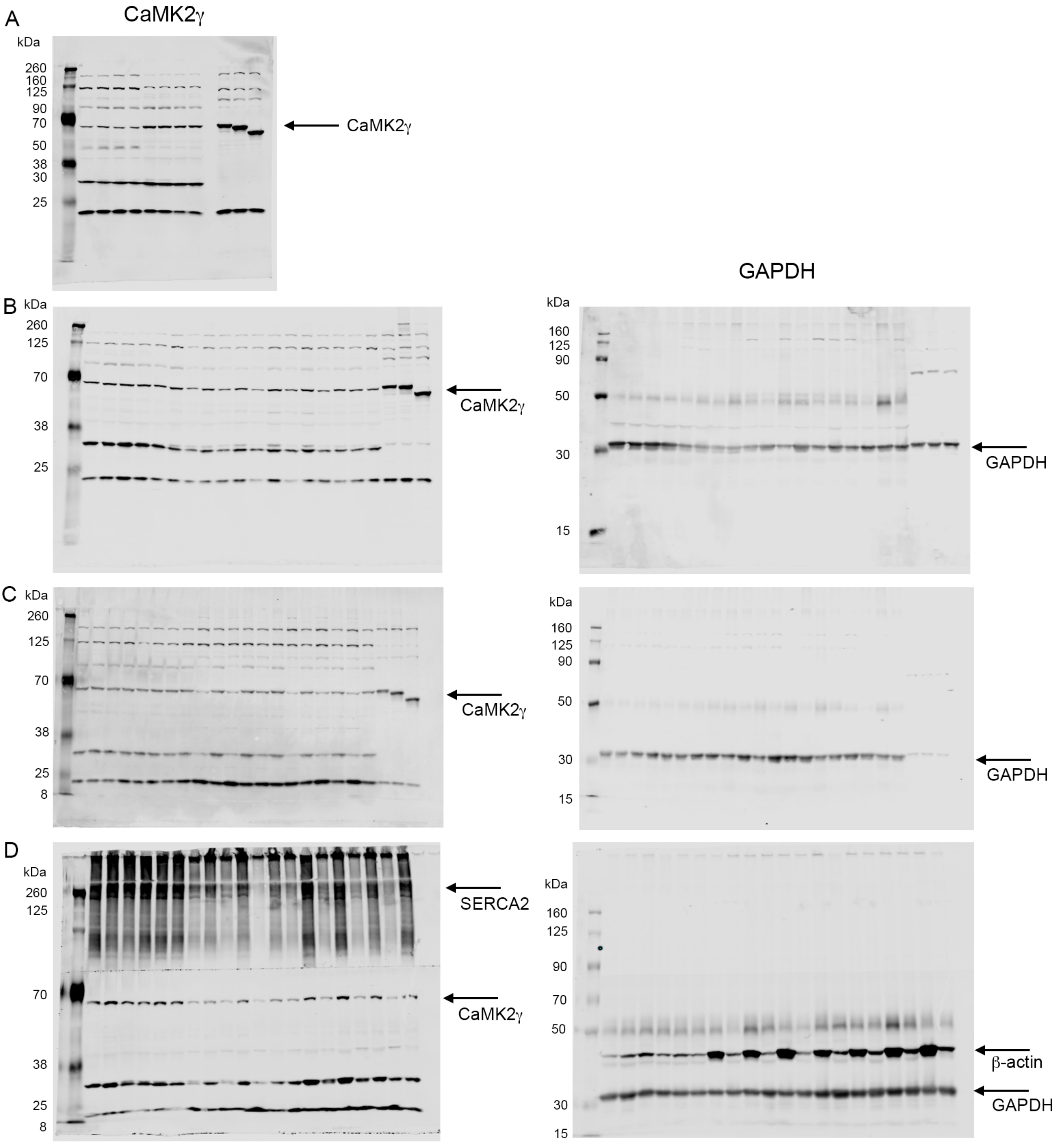

Supplementary Figure 3 continued. Uncropped western blots from Figure 4.

E

dark exposure for Camk2γ

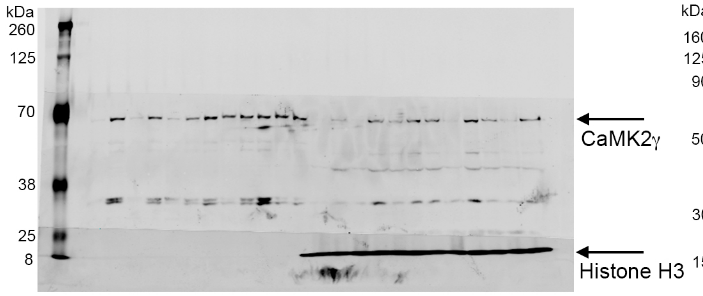

GAPDH

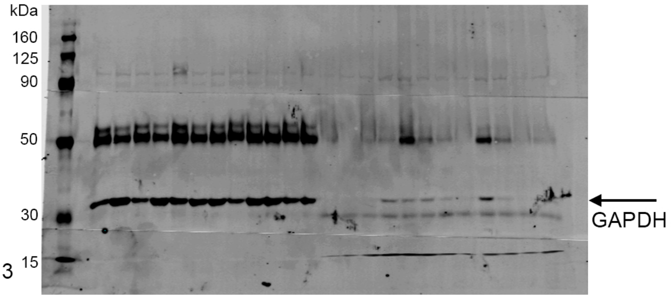

light exposure for Histone H3

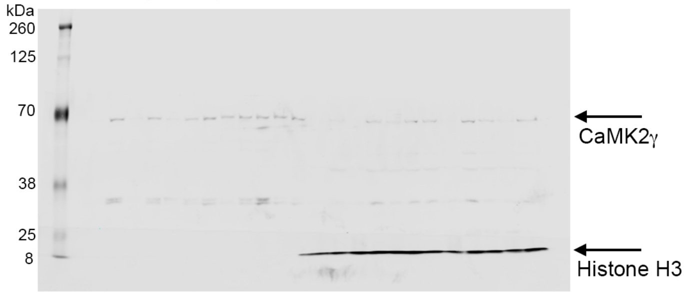

Supplementary Figure 4. Uncropped western blot from Figure 6. Panel A corresponds to Figure 6C and panel B corresponds to Figure 6D. CaMK2γ or Rbfox1 staining on left and GAPDH staining on right (indicated by arrows).

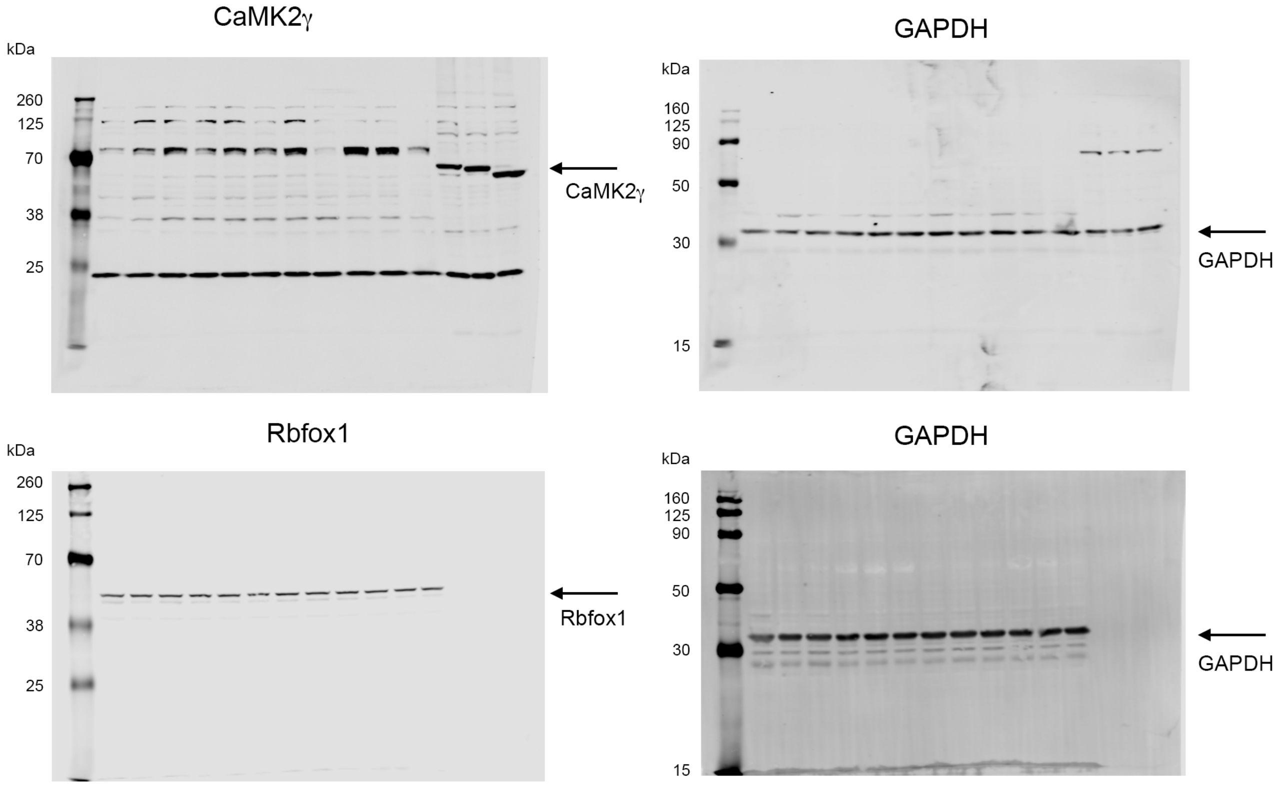

Supplement: Supplementary file 1 — Supplementary Information 1. [file 41598_2021_92426_MOESM1_ESM.pdf]
